# Interactive effects of cocaine on HIV infection: implication in HIV-associated neurocognitive disorder and neuroAIDS

**DOI:** 10.3389/fmicb.2015.00931

**Published:** 2015-09-08

**Authors:** Santosh Dahal, Sai V. P. Chitti, Madhavan P. N. Nair, Shailendra K. Saxena

**Affiliations:** ^1^CSIR-Centre for Cellular and Molecular Biology, Hyderabad, India; ^2^College of Medicine, Florida International University, Miami, FL, USA

**Keywords:** HIV, AIDS, cocaine, CNS, HAND, blood brain barrier, neuroAIDS

## Abstract

Substantial epidemiological studies suggest that not only, being one of the reasons for the transmission of the human immunodeficiency virus (HIV), but drug abuse also serves its role in determining the disease progression and severity among the HIV infected population. This article focuses on the drug cocaine, and its role in facilitating entry of HIV into the CNS and mechanisms of development of neurologic complications in infected individuals. Cocaine is a powerfully addictive central nervous system stimulating drug, which increases the level of neurotransmitter dopamine (DA) in the brain, by blocking the dopamine transporters (DAT) which is critical for DA homeostasis and neurocognitive function. Tat protein of HIV acts as an allosteric modulator of DAT, where as cocaine acts as reuptake inhibitor. When macrophages in the CNS are exposed to DA, their number increases. These macrophages release inflammatory mediators and neurotoxins, causing chronic neuroinflammation. Cocaine abuse during HIV infection enhances the production of platelet monocyte complexes (PMCs), which may cross transendothelial barrier, and result in HIV-associated neurocognitive disorder (HAND). HAND is characterized by neuroinflammation, including astrogliosis, multinucleated giant cells, and neuronal apoptosis that is linked to progressive virus infection and immune deterioration. Cocaine and viral proteins are capable of eliciting signaling transduction pathways in neurons, involving in mitochondrial membrane potential loss, oxidative stress, activation of JNK, p38, and ERK/MAPK pathways, and results in downstream activation of NF-κB that leads to HAND. Tat-induced inflammation provokes permeability of the blood brain barrier (BBB) in the platelet dependent manner, which can potentially be the reason for progression to HAND during HIV infection. A better understanding on the role of cocaine in HIV infection can give a clue in developing novel therapeutic strategies against HIV-1 infection in cocaine using HIV infected population.

## Introduction

**FIGURE 1:**
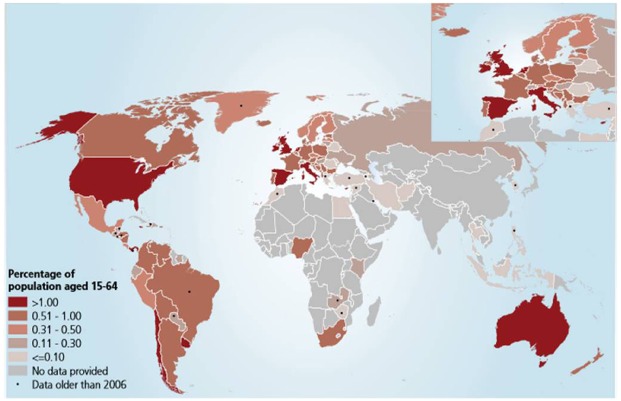
**Prevalence of cocaine use in 2010 (UNODC).** Source: UNODC estimates based on annual report questionnaire data and other official sources. The bound-aries and names shown and the designations used on this map do not imply official endorsement or acceptance by the United Nations. Final boundary between the Republic of Sudan and the Republic of South Sudan has not yet been determined. Dashed lines represent undermined bound-aries. Dotted line represents approximately the line of Control in Jammu and Kashmir agreed upon by India and Pakistan. The final status of Jammu and Kashmir has not yet been agreed upon by the parties. The final boundary between the Republic of Sudan and the Republic of South Sudan has not yet been determined.

Acquired immunodeficiency syndrome (AIDS) is a condition caused by a virus called human immunodeficiency virus (HIV). AIDS is one of the most critically acclaimed endemic diseases, caused by lentivirus HIV-1 and HIV-2 which fatally impairs the immune system (Diwan et al., 2013). According to the 2013 World Health Organization report over 35 million people were living with HIV. HIV infection and progression of AIDS can be modulated by a number of cofactors, including drugs of abuse such as opioids, cocaine, cannabinoids, methamphetamine, alcohol, and others. According to the United Nations Office on Drugs and Crime (UNODC) (2012), about 230 million people (15–64 age group, Figure 1) are estimated to have used an illicit drug and estimated number of problem drug users in 2010 was between 15.5 million and 38.6 million and more over drug users continue to be a problem with drug dependence disorders such as the prevalence of HIV (20%), hepatitis C (46.7%) and hepatitis B (14.6%). Drug abuse and addiction have been linked with HIV/AIDS since the beginning of the epidemic (Reddy et al., 2012). Substance abuse is a major barrier in eradication of the HIV outbreak the reason is that, it serves as a powerful co-factor for transmission of virus, disease progression, and AIDS related mortality. Cocaine is one of the commonly abused drugs among HIV1 patients and it has been suggested to accelerate AIDS progression. However, the principal mechanism remains largely unknown, but in recent times it was been shown that cocaine alter the behavior and mood, causing feelings of euphoria by stimulating key pleasure centers within the brain. One of the most damaging effects of cocaine abuse is that it compromises judgment capacity leading to risky sexual behavior, thereby increasing chances of contracting HIV infection. Cocaine is the second most popular abused drug in United States (Zenón et al., 2014). It is a powerfully addictive CNS stimulating drug, which increases the level of neurotransmitter DA in the brain. It has been associated with known adverse effects on cardiac, gastrointestinal tract, cerebrovascular, and pulmonary systems. Cocaine abuse increases the incidence and severity of HIV neuropathology and associated cognitive deficits by enhancing viral replication. Enhancement of AIDS pathology includes multiple immunomodulatory effects of cocaine, its capability to dysregulate neurotoxins such as quinolinic acid (QUIN) and arachidonic acid (AA) metabolites and exacerbates neurotoxicity by co-operating with viral toxins (Yao et al., 2009; Nair and Samikkannu, 2012). Additionally cocaine is also familiar as an immunosuppressant and is capable of reducing number and distribution of immune cells, white blood cells and thymocytes. The battle between human and the HIV are on, with both of them rapidly improving their attacking and defense strategies (Saxena et al., 2002).

## Role of Cocaine in Bypassing Blood Brain Barrier

Entry of HIV virus in CNS serves as the major factor for the development of the neuropathological condition and disease severity. Blood brain barrier (BBB) is the major obstacle for the entry of pathogen in CNS. Drug abuse such as cocaine disintegrates the structure and function of BBB and increases the probability of entry of pathogens into CNS. Cocaine causes BBB dysfunction through the alterations in expression and conformation of tight junction proteins such as zona occludin 1 (ZO-1) and junctional adhesion molecules 2 (JAM-2), which causes glial cell activation. This results in BBB cytoskeleton remolding through various enzymes eventually leading to neuroinflammation. Cocaine treatment additionally increases gene expression of factors, including matrix metallopeptidase-1 (MMP-1), that contributes to basement membrane actin rearrangement forming stress fiber around cerebral vessels. Persistent loss or conformational changes in tight junction proteins and reorganization of basement membrane fibers leave the brain open to peripheral toxin penetration leading to CNS disorders of cocaine misuse (Kousik et al., 2012). Apart from this cocaine has been found to increase infiltration of infected leukocytes from blood vessels into the brain through direct effect on human brain microvascular endothelial cells (HBMECs) and indirectly via release of proinflammatory cytokines such as TNF-α and IFN-γ (Wachtman et al., 2007). Exposure of HBMECs to cocaine stimulates platelet derived growth factor (PDGF) at both the transcriptional and translational levels, as binding of cocaine to its related σ 1-R receptor leads to the activation of ERK1/2 and JNK MAP kinases. This in turn triggers the release of Erg-1 transcription factor that enters the nucleus and stimulates PDGF-BB expression (Kumar, 2011). PDGF-BB is a known agent involved in disruption of BBB and is involved in cocaine-mediated decrease in ZO-1 expression involves PDGF-BB. Along with this cocaine also increases leukocyte–endothelial adhesion which is accompanied by elevated levels of pro-inflammatory cytokines and chemokines. Immunostaining and western blotting disclose increase in TNF-α, IL-6, IL-8, nuclear factor kappa B (NFκB), activator protein 1 (AP-1), and CCR2. *In vitro* BBB model showed these inflammatory signals are co-related with increased viral invasion of macrophage-trophic HIV possibly explaining greater neuropathology detected in patients co-morbid for HIV infection and cocaine abuse (Gandhi et al., 2010). This provides a picture of the effects of cocaine on BBB defined by cocaine induced neuroinflammation. Clinical studies reveal similar BBB breakdown in the basal ganglia and increased HIV penetration into the CNS of cocaine-abusing humans (Kousik et al., 2012).

## Cocaine and HIV-associated Neurocognitive Disorder

**FIGURE 2:**
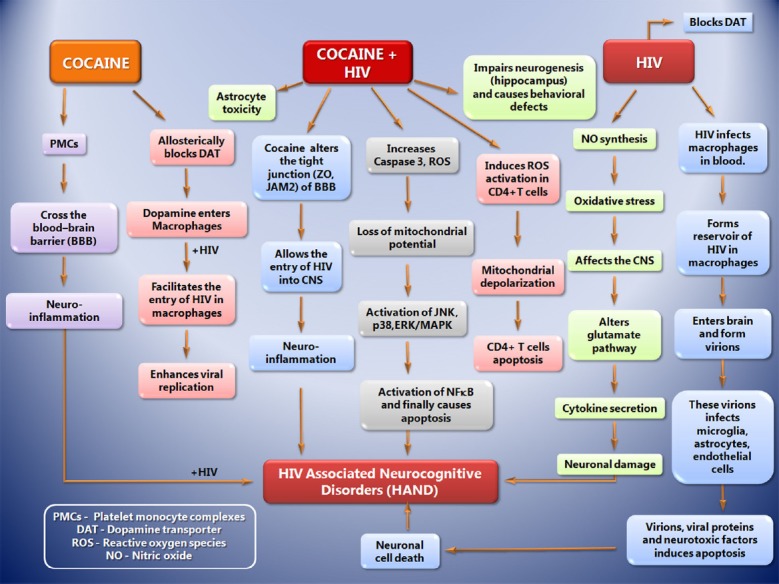
**The sequence of events that leads to progression toward HIV associated Neurocognitive Disorders (HAND) during cocaine abuse, cocaine + HIV infection and HIV infection alone.** Drug abuse (Cocaine) leads to the formation of platelet monocyte complexes (PMCs) which crosses transendothelial barrier and cause neuroinflammation, which possibly results in HAND. Along with the PMCs formation, cocaine also induces neuronal apoptosis by triggering viral products such as Tat and gp120, and potentiates astrocyte toxicity after activation by HIV gp120. gp120 is necessary for the viral infectivity, enhances neurotoxicity via inducible nitric oxide synthesis, and causes cellular oxidative stress which increasingly affects the CNS. It also alters the host glutamate pathway signaling, which interacts with cellular receptor, directing secretion of cytokines and finally affecting the neurons. Cocaine along with gp120 synergistically increases neuronal toxicity by the increase in the activity of caspase-3, increase in reactive oxygen species and loss of mitochondrial potential. Cocaine and gp120 are capable of eliciting signaling transduction pathways in neurons, involving mitochondrial membrane potential loss, oxidative stress, activation of JNK, p38, and ERK/MAPK pathways, and results in downstream activation of NF-κB that leads to HAND.

Neurologic complications may occur up to 40% of AIDS patients and nearly 25% of HIV infected persons who have not clinically progressed to AIDS (Carvour et al., 2015). During the later stages of the disease, HIV-1 infected patients suffer from a wide range of neurological and neurocognitive disorders collectively known as HIV-associated neurocognitive disorder (HAND; Atluri et al., 2014). It is characterized by neuroinflammation, including astrogliosis, multinucleated giant cells, and neuronal apoptosis that is linked to progressive virus infection and immune deterioration (Saiyed et al., 2011). Neuronal apoptosis, a result of neuronal dysfunction, stands as one of the features of HIV-associated dementia which is induced by diverse cellular and viral factors, inclusive of viral proteins Tat and gp120. Drugs of abuse act synergistically with HIV proteins to potentiate HIV related neurotoxicity (Yuan et al., 2015) and affect many functions associated with the synaptic plasticity. Introduction of the anti-retroviral therapy (ART) has decreased the cases of HAND. However, drug abuse is playing a crucial role in the prevalence of HAND in the HIV infected individuals. Cocaine use serves as a potential factor for the progression to the HAND in the HIV infected persons. One proposed mechanism is via formation of platelet monocyte complexes (PMCs). Interaction between monocytes and activated platelets leads to the formation of PMCs, and these monocytes has enhanced capability to cross trans-endothelial barrier and cause neuroinflammation, which possibly results in HAND (Tiwari et al., 2013). Along with the PMCs formation, cocaine also induces neuronal apoptosis by triggering viral products such as Tat and gp120, and potentiates astrocyte toxicity after activation by HIV gp120 (Yang et al., 2010). gp120 is necessary for the viral infectivity, enhances neurotoxicity via inducible nitric oxide synthesis, and causes cellular oxidative stress which increasingly affects the CNS (Figure 2). It also alters the host glutamate pathway signaling, which interacts with cellular receptor, directing secretion of cytokines and finally affecting the neurons (Samikkannu et al., 2011). Cocaine along with gp120 has been shown to synergistically increase neuronal toxicity. This has been defined by the increase in the activity of caspase-3, increase in reactive oxygen species and loss of mitochondrial potential. Studies have also suggested that cocaine and gp120 alone are capable of eliciting signaling transduction pathways in neurons, involving mitochondrial membrane potential loss, oxidative stress, activation of JNK, p38, and ERK/MAPK pathways, and results in downstream activation of NF-κB (Yao et al., 2009). *In vitro* studies have shown that neuronal toxicity has been found to be associated with increased cathepsin B secretion in MDM with HIV infection along with cocaine treatment (Zenón et al., 2014).

## Effect of Cocaine and HIV Proteins on Dopamine Transporter

**FIGURE 3:**
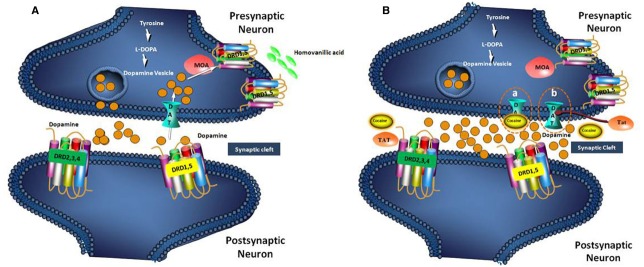
**Mechanism of Dopamine reuptake by DAT receptor (A) and blocking of the DAT receptor by cocaine and HIV Tat protein (B). (A).** Dopamine, transmits brain signals by flowing from presynaptic neuron into the synaptic cleft and attaching to a receptor (DRD 1,5, and DRD 2,3,4) on postsynaptic neuron. In general, excess dopamine is recycled into the presynaptic neuron by a dopamine transporter (DAT) on the surface of the presynaptic neuron. The recycled dopamine is then degraded into homovanillic acid via monoamine oxidase (MOA). **(B-a)**. In this case of cocaine drug abuse, the drug (cocaine) attaches to the DAT and blocks the normal recycling of dopamine, causing an increase of dopamine levels in the spaces between neurons that lead to inflammation. **(B-b)**. The dopamine recycling is also inhibited by the Tat protein of HIV by bringing about conformational changes in DAT. The high concentration of dopamine in the synaptic cleft causes inflammation.

Dopamine is a neurotransmitter that is produced from amino acid tyrosine and belongs to the catecholamine family. DA synthesis occurs in the cytoplasm of the neuron and then it is transported within the secretary vesicles of the neuron for storage and subsequent discharge. Once put away at nerve terminals, DA is prepared to be released into the synaptic cleft and subsequently, a greater part of it is recaptured by the dopamine transporter (DAT), which is present in the pre synaptic membrane (Figure 3A, Giros et al., 1992; Rudnick, 2011). The high concentrations of DA at synaptic cleft causes apoptosis or inflammation to the tissue. There are three main pathways controlled by DA, firstly the pathway of the nigro striatal system, secondly the forebrain that includes cerebral cortex, nucleus accumbens and other limbic structure and finally tubero-infundibular pathway, in which DA release occurs directly in the portal system.

Dopamine dysregulation has been associated with HAND, and evidence has implicated brain regions rich in DA are highly susceptible to the effects of HIV (Jacobs et al., 2013). The DAT is critical for DA homeostasis, which is important for neurocognitive function and this is the target for HIV viral proteins and cocaine. Tat acts as an allosteric modulator of DAT, where as cocaine acts as reuptake inhibitor (Figure 3B). The DAT serves as the primary mechanism for regulating dopaminergic tone in the synapse and it is directly affected by HIV-1 infection and cocaine. This in turn intensifies viral replication and Tat release, resulting in compromised DA function and the manifestation of HAND (McIntosh et al., 2015).

Macrophages hold a major part in HIV mediated neuropathogenesis and may function as viral reservoirs within the CNS. Macrophages are also found to release inflammatory mediators and neurotoxic viral and host proteins, causing chronic neuroinflammation. Thus, HIV infection of infiltrated macrophages of CNS is critical to HIV-associated neurocognitive dysfunction and neuroinflammation. Macrophages in the CNS are exposed to DA, which is increased by illicit drugs such as cocaine and methamphetamine. Study showed that DA at concentrations above 10^–8^ M caused approximately twofold increase in HIV entry into human primary monocyte-derived macrophages (Gaskill et al., 2014).

During the synergistic effect of HIV infection and cocaine abuse, the levels of DA increases in the synaptic cleft. The studies showed that DA increases HIV replication in human macrophages through activation of DA receptor, increasing the total number of infected cells. The mechanism(s) by which this occurred are unclear, but one possibility is by increasing HIV entry into macrophages. HIV entry is complex, and in macrophages, it is mediated by the interaction of the viral envelope protein gp120 with the surface receptor cluster of differentiation 4 (CD4) and CCR5 on the macrophages (Sundaravaradan et al., 2006) or through alternative pathways such as the endocytic pathway or through interaction with the co-receptor CXCR4 or minor co-receptors including CCR3 (Peters et al., 2004). DA is able to modulate macrophage functions because macrophage expresses DA receptors.

## Cocaine and Tat Protein Impairs Neurogenesis

Mounting evidence specifies that along with neuronal loss on the brains of patients with HIV-associated CNS disease, there is also evidence of smaller number of neural stem/cells (NPCs) in the dentate gyrus of the hippocampus. There occurs continuous generation of new dentate granule from neural progenitor cells, which gets integrated into the existing hippocampal circuitry in the adult mammalian brain by a process termed as neurogenesis (Yao et al., 2012). HIV trans-activating protein Tat, which is released by infected cells and also taken up by neighboring cells has been shown to impair neurogenesis (Mishra et al., 1989). In addition to this, cocaine also diminishes the proliferative rate of NPCs by negatively affecting the self-renewal capacity of the hippocampus (Yao et al., 2012). Such defect could explain the reason for increased prevalence of behavioral deficits detected in patients with HAND in the post-ART era.

## Role of CD4^+^ T-cell and PBMC in Cocaine Associated HIV Infection

The entry of HIV into cells requires the interaction of the viral exterior envelope glycoprotein gp120 with the CD4 receptor, which is expressed on the surface of the T cells, monocytes, macrophages and dendritic cells. The interaction of CD4 and gp120 allows the fusion of viral and host cellular membranes (Collman et al., 1990; Kwong et al., 1998). The intake of HIV-1 by antigen presenting cells (APCs) and successive transfer of virus to CD4^+^ T cells can bring about significant levels of virus replication in the T cells (Rinaldo, 2013). Several studies have shown that cocaine abuse during HIV infection promotes the viral replication in CD4^+^ T cells and progression to AIDS. It was also suggested that CD4^+^ T-cell counts is lower in cocaine-abusing HIV patients in comparison to no abusers patients along with augmented decline in CD4^+^ T cell count (Chaisson et al., 1989; Anthony et al., 1991; Cook, 2011; Rafie et al., 2011). The effect of the cocaine abuse in HIV infected individuals can be preceded by the *in vitro* study showing that cocaine along with the HIV virion induces the apoptosis in the CD4^+^ T cell (Pandhare et al., 2014). Apoptosis was merely the result of the increment of the reactive oxygen species and induction of the mitochondrial depolarization by cocaine.

Apart from these, it has also been shown that micro RNA miR125b has a prime role in eliciting anti-HIV role and decreases HIV replication in CD^+^4 T cells. 3′UTR region of HIV-1 is the target for miR-125b and inhibit viral protein translation. Cocaine in such individuals reduces the expression of miR125b and promotes viral replication (Mantri et al., 2012). Integration is the important part in the replication of the virus in host cells. Cocaine in such case has been found to promote HIV-1 integration in host genome (Addai et al., 2014). Underlying mechanism is revealed as the consequence of induction of global DNA de-methylation in CD4^+^ T cells, which is concurred with the suggestion that DNA methylation disfavor integration of HIV-1. Cocaine has been found to differentially regulate the expression of numerous key host proteins that perhaps have an influence on HIV-1 replication, when proteomics analysis was done for cocaine-treated peripheral blood mononuclear cells (PBMCs) collected from HIV positive individuals. Cocaine has been shown to boost HIV-1 replication in immune cells as well as dendritic cells, macrophages and PBMCs (Reynolds et al., 2009).

## Future Perspective

Cocaine is one of the commonly abused drugs among HIV/AIDS patients and it has been suggested to accelerate AIDS progression. Cocaine not only increases the migration of peripheral HIV-infected leukocytes into CNS, but also modulates the disease status and possibility result in the neuroAIDS. A better understanding of DA and DAT role may give a clue in cocaine and AIDS progression. The mechanism of cocaine drug abuse and neuroAIDS will probably require knowledge of the interrelationship of the different variables, rather than concentrating on certain particular zones. Further study is required to advance the research toward the identification of new methodologies for the novel drug delivery system that can pass through the BBB and treat neuroAIDS. Additional research is needed to build upon from what has already been known, for this the scientists from neurobiology and infectious diseases should work collaboratively to conduct the preclinical studies and every other pathway has to be examined. A co-ordinate effort is required by researchers, doctors, drug makers, policy makers, government bodies nationally, and all around to combat the neuroAIDS. Emphasis should be laid on rehabilitation of cocaine abusers so that the chances of disease progression can be halted. Considering the seriousness of the diseases, it’s imperative to be advised to bring information about HAND into HIV prevention and awareness programs, particularly to encourage early diagnosis and treatment.

### Conflict of Interest Statement

The authors declare that the research was conducted in the absence of any commercial or financial relationships that could be construed as a potential conflict of interest.
